# The Potential Antioxidant Activity and Characterization of Bioactive Compounds of *Stahlianthus involucratus*

**DOI:** 10.1155/2021/9490162

**Published:** 2021-08-24

**Authors:** Wengkun Li, BingMin Wu, Yange Wang, Ying Lin, Lin An, Guifang Zhang

**Affiliations:** School of Pharmaceutical Science, Guangzhou University of Chinese Medicine, Guangzhou, Guangdong 510006, China

## Abstract

*Stahlianthus involucratus* (*S. involucratus*) has anti-inflammatory, antinociceptive, and antipyretic activities; however, there are no literature reports on its antioxidant capacity. This study presents a comparative assessment of the polyphenols contents, flavonoids contents, and antioxidant activity of the aqueous and methanol extracts of *S. involucratus* (ASI and MSI). Moreover, the expression of oxidative stress-related genes in H_2_O_2_-induced H9c2 cells pretreated with the MSI was measured by RT-qPCR, and furthermore, MSI were characterized by UHPLC-Q-Orbitrap-MS/MS. The results indicated that the MSI had higher antioxidant contents and antioxidant capacity, and MSI could inhibit H_2_O_2_-induced oxidative stress in H9c2 cells by activating the Nrf2/HO-1 pathway. UHPLC-Q-Orbitrap-MS/MS characterized 15 phenolic compounds from the MSI. In conclusion, *S. involucratus* has the potential antioxidant capacity.

## 1. Introduction

*S. involucratus*, belonging to Zingiberaceae [1], as a Traditional Chinese Medicine (TCM), was mainly used for the treatment of bruises and rheumatic arthralgia [2]. The plants in Zingiberaceae family were enriched with bioactive compounds; the previous reporters had confirmed the phytochemical and pharmacological properties of Zingiberaceae [3]. The ethanol extracts of *S. involucratus* had anti-inflammatory, antinociceptive, and antipyretic activities [4, 5]. The extraction of *Elettaria cardamomum*, *Curcuma longa*, *Zingiber officinale*, and *Alpinia officinarum* had the scavenging activity of the cationic ABTS radical and ferric-reducing antioxidant power-FRAP [6]. The standardized extraction from the rhizomes of ginger defended a rat liver from cancer by decreasing oxidative and inflammatory injury [7]. The reporter verified that cardamonin from the Alpinia plant inhibits oxidative stress, apoptosis, and inflammatory responses in the mouse heart by improving NRF2 signaling [8]. Excessive secretion of free radicals causes oxidative damage to cells and tissues, induces oxidative stress, and further leads to inflammatory responses and damages the immune system [9]. Nuclear factor-related gene 2 (NRF2), a transcription factor, is thought to be the dominant factor in the regulation of cellular oxidative stress [10]. The activated NRF2 binds to antioxidant response elements (ARE) to regulate downstream genes and then defend against damage caused by oxidative stress.

Oxidative stress, a negative effect of free radicals in the body, is associated with various diseases, including inflammatory response, cell damage, tissue sclerosis, and cancer [11]. Natural plant extracts are rich in bioactive components such as phenols, flavonoids, and glycosides. Modern research has demonstrated that all these compounds have antioxidant, anti-inflammatory, and antitumor activities and can act directly on inflammatory and tumor cells without affecting the viability of normal cells [12, 13].

This study presents a comparative assessment of the polyphenols contents, flavonoids contents, and antioxidant activity of ASI and MSI, and furthermore, the expression of *Nqo1*, *Ho-1*, *Gclc*, *Gclm*, *Gst*, and *Nfe212* in H_2_O_2_-induced H9c2 cells pretreated with the MSI was examined by RT-qPCR, and then the phytochemical compounds from MSI were isolated and characterized by UHPLC-Q-Orbitrap-MS/MS.

## 2. Materials and Methods

### 2.1. Chemicals and Reagents

Folin's reaction solution, sodium carbonate, 2,4,6-tripyridyltriazine (TPTZ), 2′-azino-bis-(3-ethylbenzthiazoline-6-sulphonate) (ABTS), 2,2-diphenyl-1-picrylhydrazyl (DPPH), potassium persulfate, quercetin, trolox, gallic acid, FeCl_3_, sodium acetate, and AlCl_3_ were from Macklin Biochemical Co., Ltd. (Shanghai, China). Methanol and acetonitrile were purchased from Thermo Fisher Scientific (MA, USA), and formic acid was purchased from Aladdin Biochemical Technology Co., Ltd. (Shanghai, China). Distilled water was obtained from a distilled water machine (EPED-E1-10TJ).

*S. involucratus* (King ex Bak.) Craib was collected from Yangshan County, Qingyuan City, Guangdong Province, China.

### 2.2. Extraction Methods

#### 2.2.1. ASI Preparation

Thirty grams of dried powder with 600 mL distilled water was decocted at 100°C for half an hour in a water heater, and the mixture was then filtered with a gauze. The supernatant was obtained by centrifugation. Repeat the above extraction procedure. The supernatants consolidated were dried by a freeze-drying method and stockpiled in the refrigerator at -20°C.

#### 2.2.2. MSI Preparation

Thirty grams of dried powder with 600 mL methanol was extracted for an hour in an ultrasonic bath, and the mixture was then filtered with a filter paper. Repeat the above extraction procedure. The consolidated supernatants were concentrated and dried by a freeze-drying method and stockpiled in the refrigerator at -20°C.

### 2.3. Physicochemical and Antioxidant Analysis

#### 2.3.1. Estimation of Total Phenolic Content (TPC)

The solution, a mixture of 25 *μ*L of the sample solution and 25 *μ*L of Folin's reagent, was kept at 25°C for 5 min in the dark, and next, 25 *μ*L of 10% (*w* : *w*) sodium carbonate solution and 200 *μ*L distilled water were added. The absorbance was detected at 765 nm using a UV spectrophotometer. The total phenolic content was expressed as *μ*g gallic acid equivalents per gram extract (*μ*g GAE/mg of extract).

#### 2.3.2. Estimation of Total Flavonoid Content (TFC)

The solution, a mixture of 80 *μ*L of the sample solution, 80 *μ*L 2% (*w* : *w*) aluminum chloride solution, and 100 *μ*L sodium acetate (50 g/L), was kept at 25°C for 2.5 h in the dark. The absorbance was detected at 415 nm using UV spectrophotometer. The total flavonoid content was expressed as *μ*g quercetin equivalents per gram extract (*μ*g QE/mg of extract).

#### 2.3.3. ABTS Analysis

The ABTS reaction solution consisting of the potassium persulfate (0.7 mg/L) and the ABTS (4 mg/L) at 1 : 1 was kept at 25°C for 18 h in the dark. The solution, a mixture of 10 *μ*L of the sample extraction and 290 *μ*L of ABTS reaction solution, was added to 96-well plates and incubated at 25°C for 15 min. The absorbance was detected at 734 nm using a UV spectrophotometer. The results were expressed as *μ*g ascorbic acid equivalents per gram extract (*μ*g AAE/mg of extract).

#### 2.3.4. DPPH Analysis

The solution, a mixture of 40 *μ*L of the aqueous extraction and 260 *μ*L of DPPH reaction solution (40 mg/L), was added into the plates and incubated at 25°C for 30 min. The absorbance was detected at 517 nm using a UV spectrophotometer. The results were expressed as *μ*g ascorbic acid equivalents per gram extract (*μ*g AAE/mg of extract).

#### 2.3.5. FRAP Analysis

The FRAP solution consists of 10 mM TPTZ solution, 20 mM ferric chloride solution, and 0.3 M of sodium acetate solution at 1 : 1 : 10. The solution composing of 20 *μ*L of the sample extract and 280 *μ*L of FRAP solution was kept at 37°C for 10 min. The absorbance was detected at 593 nm using a UV spectrophotometer. The results were expressed as *μ*g ascorbic acid equivalents per gram extract (*μ*g AAE/mg of extract).

### 2.4. Cell Culture

The H9c2 cell line was purchased from the Cell Bank of Chinese Academy of Sciences (Shanghai, China). The H9c2 cells were cultivated within the DMEM (Thermo Fisher Scientific, MA, USA) containing 10% fetal bovine serum (FBS, Thermo Fisher Scientific, MA, USA), 100 *μ*g/mL streptomycin, and 100 U/mL penicillin. Afterward, cells were subjected to incubation under 37°C and 5% CO_2_ conditions. When cells reached confluence, PBS was used to wash cells for two times and the serum-free medium was used for further cell culture prior to the subsequent experiment.

### 2.5. Cell Viability

The cell viability was determined by the MTT method. In 96-well plates, the cells were grown in the complete medicated DMEM medium (0, 1, 5, 25, 50, 100, 250, 500, and 1000 *μ*g/mL of MSI) at 37°C with 5% CO_2_ for 24 h, 10 *μ*L MTT (5 mg/mL) was added into the plates; next, the plates were incubated for 4 h. Adding the 150 *μ*L DMSO after the medium was waived. The microplate reader (Thermo Varioskan LUX, MA, USA) was used to measure the absorbance (OD) value at 490 nm.

### 2.6. Assay of ROS Production

Cultured H9c2 cells were treated with indicated agents for 16 h, and the intracellular ROS generation was determined by measuring the oxidative conversion of cell permeable DCFH-DA to fluorescent dichlorofluorescein. Images were viewed by a fluorescence microscope (Leica, Heerbrugg, Germany) at an excitation wavelength of 488 nm and an emission wavelength of 525 nm.

### 2.7. Total RNA Isolation and Quantification

In real-time PCR experiment, the cells were seeded in a 6-well plate and the optimal cell number is 1 × 10^6^ cells per well. H9c2 cells were incubated with 200 *μ*M H_2_O_2_ at 37°C with 5% CO_2_ for 4 h and then incubated the complete medicated DMEM medium (25, 50, and 100 *μ*g/mL of MSI) at 37°C with 5% CO_2_ for 12 h. Total RNA was obtained by a Trizol method; further the purity and concentration of total RNA were determined. The expression of target genes was measured using Real-time Quantitative PCR (RT-qRCR) after the total RNA was reverse-transcribed to cDNA. The housekeeping gene was *Gapdh* gene. The sequences of the target genes are listed in Table 1. The results were analyzed using the 2^-*ΔΔ*Ct^ method.

### 2.8. Metabolomics Profiling by UHPLC-Q-Orbitrap-MS/MS Technique

In the present literature, the compounds from S. Involucratus, including ergotane dimer, sesquiterpenes, cardanane dimer, and alkaloids, were isolated and characterized by UPLC-MS [14, 15]. Ultrahigh performance liquid chromatography (UHPLC) coupled with an electrospray ionization tandem mass spectrometry (ESI-MS-MS) is a common technique for the isolation and identification of natural products [16]. The Q-Orbitrap, a high-resolution mass spectrometer, allows molecular weight and fragment information. UHPLC-Q-Orbitrap-MS/MS are used to identify the metabolites in herbal medicines [17, 18]. The compounds in TCM were characterized by combining parent ions with fragment ions. These means were extensively applied to phytochemical analysis as a result of accuracy, efficiency, and celerity.

MSI were analyzed by UHPLC-MS/MS spectrum consisting of Thermo Scientific™, Ultimate™ 3000RS, Thermo Scientific™, Q Exactive™, Hbrid Quadrupole-Orbitrap mass spectrometer, ESI source, and RP-C18 column (150 × 2.1 mm, 1.8 *μ*m). The column temperature was at 35°C. Mobile phase A was the solution of aqueous containing 0.1% formic acid, and Mobile phase B was the solution of acetonitrile containing 0.1% formic acid. The elution procedure (A : B (*v*/*v*) at time (min)) was as follows: (98 : 2) at 0 min; (98 : 2) at 1 min; (80 : 20) at 5 min; (50 : 50) at 10 min; (20 : 80) at 15 min; (5 : 95) at 20 min; (5 : 95) at 25 min; (98 : 2) at 26 min; and (98 : 2) at 30 min, and the injection volume was 5 *μ*L. The condition of the mass spectrometer was as follows: scan range 150–2000 *m*/*z*; aux gas heater temperature 350°C; capillary temperature 300°C; spray voltage 3.8 kV; and sheath gas pressure 40 Arb. The aux gas and sheath gas were high purity nitrogen gas (purity ≥ 99.999%), and the collision gas was high purity argon gas (purity ≥ 99.999%). Full-mass and dd-MS^2^ data in positive and negative modes were acquired at 70,000 and 17,500 FWHM (full width half maximum), respectively.

### 2.9. Statistical Analysis

The results were tested by one-way analysis of variance (ANOVA); *P* < 0.05 was regarded as significant and evaluated with Tukey's test by SPSS 20.0 software. The data acquired by UHPLC-MS/MS spectrum was processed by CD2.1 (Thermo Fisher Scientific), and then the compounds were characterized according to precursor ions and fragment ions compared with the chemical database like mzCloud, mzVault, and ChemSpide.

## 3. Results and Discussion

### 3.1. Estimations of TPC and TFC

The antioxidant capacity can be roughly determined by binding estimations of polyphenols and antioxidant assays. The aluminum trichloride method and Folin-Ciocalteu method were currently used for the determination of total flavonoids and total phenols, and the results of TFC and TPC were expressed as quercetin equivalents (QE)/g of dried extraction and gallic acid equivalents (GAE)/g dried extraction, respectively.  Table 2 points out that the TPC and TFC of the MSI were 110.24 ± 1.01 mg GAE/g dried extraction and 12.30 ± 0.10 mg QE/g dried extraction, respectively, and were significantly higher than those of the ASI.

### 3.2. Antioxidant Activities (DPPH, ABTS, and FRAP)

There were many experiments to evaluate antioxidant activity in vitro. In this study, the DPPH, ABTS, and FRAP assays were used to validate the antioxidant activity of *S. involucratu*s. Both DPPH and ABTS assays were detected by binding antioxidants to colored ionic free radicals, causing the color to fade. However, the FRAP assay was detected by the reduction of chelated ions by antioxidants, resulting in a color reaction. In short, a comprehensive analysis of the three assays can determine initially the antioxidant capacity. As described in Table 2, in the ABTS, DPPH, and FRAP assays, the result showed that the free radical scavenging capacity of the MSI was 89.62 ± 1.65, 21.09 ± 0.34, and 30.60 ± 0.27 mg TE/g dried extraction, respectively, and was higher than that of the ASI (*P* < 0.05), which indicated that the MSI had stronger antioxidant activity.

The results were expressed as the means ± SD (*n* = 3). Different letters show significant differences in the extracts by different methods (*P* < 0.05).

### 3.3. The MSI Activated Nrf2/HO-1 Pathway after H_2_O_2_-Induced H9c2 Cell

**Figure 1:**
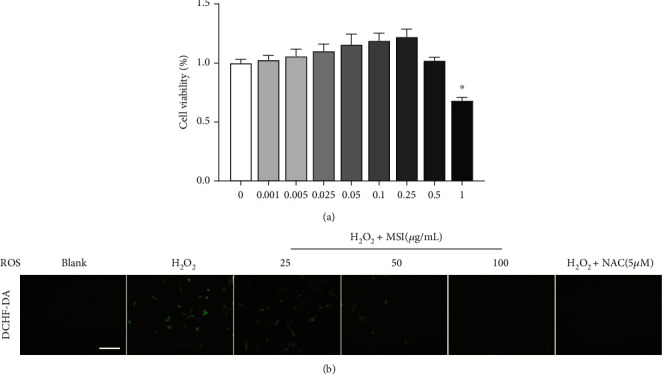
The cell viability and the mRNA expression of related genes. (a) The cell viability of MSI. (b) The ROS levels. The results were expressed as the mean ± standard deviation (*n* = 3); ^∗^*P* < 0.05, versus the control. “blank” means the “control untreated cells”.

First, the cell viability was assessed and MSI showed no toxicity at the concentration ranging from 0.001 to 1 mg/mL, excluding the possibility that MSI influenced phenotypes *via* cytotoxicity (Figure 1(a)). Further, we evaluated the effects of MSI on ROS production by determining the ROS levels *in vitro* using commercial assay kit (Figure 1(b)). As anticipated, MSI effectively reduced ROS generation in cardiomyocytes, demonstrating its oxidative stress suppressing action.

**Figure 2:**
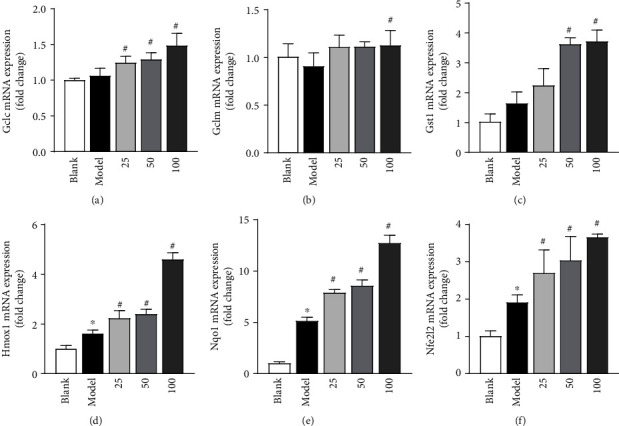
The mRNA expression of related genes. (a) The mRNA expression of *Gclc*. (b) The mRNA expression of *Gclm*. (c) The mRNA expression of *Gst*. (d) The mRNA expression of *Hmox-1*. (e) The mRNA expression of *Nqo1*. (f) The mRNA expression of *Nfe212*. The results were expressed as the mean ± standard deviation (*n* = 3); ^∗^*P* < 0.05, versus the control; ^#^*P* < 0.05, versus the model group. “blank” means the “control untreated cells”; “model” means “the cells pretreated with H_2_O_2_”.

ROS are the driving force for myocardial damage, and Nrf2 is a key orchestrator of the cell responses to oxidative stress and exerts a protective effect against oxidative damage. According to this, the expression of *Nqo1*, *Ho-1*, *Gclc*, *Gclm*, *Gst*, and *Nfe212* was measured after H_2_O_2_-induced H9c2 cells treated with different concentrations of the MSI (25, 50, and 100 *μ*g/mL) and shown in Figure 2(a)–2(f). For *Gclc*, *Ho-1*, *Nqo1*, and *Nfe212* mRNA, the expression level was significantly increased in the administration group (25, 50, and 100 *μ*g/mL) compared to the model group (*P* < 0.05). Furthermore, compared with the model group, 100 *μ*g/mL of MSI significantly increased the expression of *Gclm*, and 50 and 100 *μ*g/mL of MSI significantly increased the expression of *Gst* (*P* < 0.05).

### 3.4. UHPLC–MS/MS Analysis of the MSI

**Figure 3:**
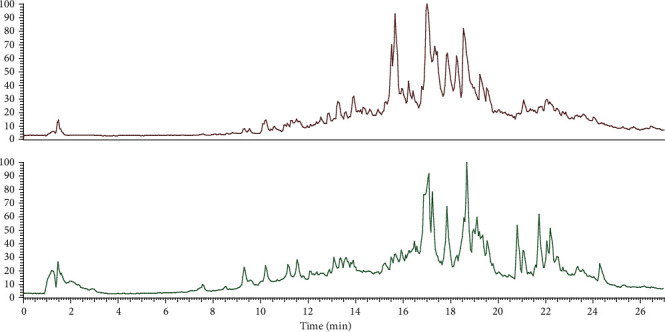
The positive and negative ion chromatogram of MSI of *S. involucratus*. The red line represents positive ions; the green line represents negative ions.

The metabolites were characterized by UHPLC-Q-Orbitrap-MS/MS under negative ion collection modes. The total ion chromatogram of *S. involucratus* is shown as Figure 3. Fifteen compounds, whose score was higher than 80, were tentatively identified, and the relevant information is presented in Table 3.

A total of 13 phenols were tentatively detected. Peak **1** was identified as 4-pyridoxic acid at *m*/*z* 182.0450 [M-H]^−^. O-Desmethyl-cis-tramadol (peak **2**) and 4-coumaric acid (peak **7**) were characterized at *m*/*z* 250.1801 and 165.0547, respectively, in ESI^+^. Peaks **5**, **8**, and **13**, belonging to phenolic acid, were tentatively identified as vanillic acid, ferulic acid, and (8-hydroxy-4a,8-dimethyldecahydro-2-naphthalenyl) acrylic acid with [M-H]^−^ at *m*/*z* 167.0839,193.0496, and 251.1648. As described in Table 3, peaks **3**, **4**, **6**, and **9** were assigned as 2-(hydroxymethyl)-6-[(E)-4-(1,2,4-trihydroxy-2,6,6-trimethylcyclohexyl) but-3-en-2-yl]oxyoxane-3,4,5-triol, 3-[2-(*β*-D-glucopyranosyloxy)-4-methoxyphenyl]propanoic acid, 1,2,3,6-tetra-O-galloyl-*β*-D-glucose, and 4-[4-(4-hydroxy-3-methoxyphenyl)-tetrahydro-1H,3H-furo[3,4-c]-furan-1-yl]-2-methoxyphenyl hexopyranoside at *m*/*z* 451.2184, 357.1188, 787.0996, and 565.1692, respectively, in ESI^−^ mode. In addition, in the negative mode, 1,7-bis(4-hydroxyphenyl)-3,5-heptanediol (peak **11**), 1-naphthol (peak **14**), and curcumin (peak **15**) were characterized at *m*/*z* 315.1599, 143.0489, and 385.129. Furthermore, 2 flavonoids, peaks **10** and **12**, were assigned as astragalin and afzelin, which were at *m*/*z* 465.1044 and 431.0919 [M-H]^−^.

## 4. Discussion

Oxidation is closely related to human diseases, and oxidation-induced damage is the most fundamental pathological process of the disease, so the exploration of natural antioxidants is vital [19]. Phenols, as natural antioxidant substances, scavenge free radicals by blocking the transmission process of free radicals and inhibit oxidative reactions by indirectly preventing the production of oxidative radicals complexing enzymes related to free radicals or metal ions [20]. This experiment provided a preliminary cognition of the antioxidant properties of *S. involucratus*. In the comparison of the total polyphenols and total flavonoid contents of the ASI and MSI, the results showed that there are higher total polyphenol and total flavonoid contents in the MSI. Besides, in the ABTS, DPPH, and FRAP assays, the MSI has stronger free radical scavenging power and reduction capacity than the ASI.

In addition, the extracts of natural products effectively inhibit cell damage by suppressing oxidative stress [21–23], and the Nrf2/HO-1 signaling pathway plays an important role in mitigating oxidative stress damage to cells and tissues [24], and HO-1 is one of the important endogenous protective enzymes of downstream of the Nrf2/HO-1 pathway [25], and the expression of HO-1 can inhibit oxidative reactions and thus protect cells and tissues from free radical damage [26]; the modern research had shown that natural product extracts are effective against H_2_O_2_-induced oxidative stress by activating Nrf2/HO-1 pathway [27, 28]. In this study, MSI effectively reduced ROS generation in cardiomyocytes, demonstrating its oxidative stress suppressing action, and the expression of the regulatory genes (*Nfe212*, *Hmox-1*, and *Nqo1*) has increased significantly after H_2_O_2_-induced H9c2 cells pretreated with MSI; furthermore, the expression levels of Gclm, Gclc, and Gst mRNA have elevated. These results indicated that the MSI could inhibit oxidative stress by activating the Nrf2/HO-1 pathway.

Furthermore, the biochemical compounds of MSI were identified by liquid chromatography-mass spectrometry (LC-MS). LC-MS technology plays a major role in the characterization of the complex natural product [29]. Ultraperformance liquid chromatography-mass spectrometry coupled with Orbitra is more rapid and accurate for the isolation and identification of the metabolites of herbal medicines [30]. In this study, 15 phenolic compounds were characterized, including vanillic acid, 4-coumaric acid, ferulic acid, astragalin, afzelin, and curcumin. In the previous studies, vanillic acid, a bioactive phenolic compound, was proven to antioxidant and inhibit oxidative stress [31–33], which have anti-inflammatory and neuroprotective effects [34, 35]. In addition, the plant phenolic acid 4-coumaric acid protected against oxidative DNA damage in rat colonic mucosa and ultraviolet B-induced oxidative DNA damage in rabbit corneal-derived cells [36, 37]. Ferulic acid exhibits a variety of pharmacological properties, such as antioxidant [38, 39], ameliorating the pulmonary fibrosis [40], alleviating the acute liver injury [41] and lipid-lowering effect [42]. Astragalin and afzelin were the natural flavonoids and found in various herbal medicinal plants such as *Cuscuta chinensis* [43]; modern pharmacological researches have shown that astragalin carried out antioxidant and anti-inflammatory activity through the regulation of NO, malondialdehyde (MDA), reactive oxygen species (ROS), superoxide dismutase (SOD), glutathione peroxidase (GSH-Px), catalase (CAT), tumor necrosis factor-*α* (TNF-*α*), interleukin 6 (IL6), and inducible nitric oxide synthase (iNOS) [44, 45]. Similarly, afzelin acts as the oxidants to scavenge free radicals, superoxide anion, and reactive oxygen species [46]. Additionally, curcumin is widely found in the Zingiberaceae family plants, such as *C. longa* [47], which is well known for its numerous pharmacological properties, including anti-inflammatory, antioxidant, neuroprotective, antiobesity, antiosteoporosis, anticancer, and antidiabetic properties [48–51].

## 5. Conclusion

In short, the results demonstrated that the antioxidants contents of MSI were more abundant than those of ASI, and the MSI inhibits H_2_O_2_-induced oxidative stress in H9c2 cells by activating the Nrf2/HO-1 pathway. These findings give guidance for the clinical use of *S. involucratus.*

## Figures and Tables

**Table 1:** The primer sequences of *Nqo1*, *Ho-1*, *Gclc*, *Gclm*, *Gst*, and *Nfe212*.

Primers	Primer sequence
*Nqo1*-F	5′-GGCCATCATTTGGGCAAGTC-3′
*Nqo1*-R	5′-TCCTTGTGGAACAAAGGCGA-3′
*Ho-1*-F	5′-GTAAATGCAGTGTTGGCCCC-3′
*Ho-1*-R	5′-ATGTGCCAGGCATCTCCTTC-3′
*Gclc*-F	5′-GAGCGAGATGCCGTCTTACA-3′
*Gclc*-R	5′-TTGCTACACCCATCCACCAC-3′
*Gclm*-F	5′-ATGGAGCTCCCAAATCAGCC-3′
*Gclm*-R	5′-CCACTGCATGGGACATGGTA-3′
*Gst*-R	5′-GCTGGAGTGGAGTTTGAAGAA-3′
*Gst*-F	5′-GTCCTGACCACGTCAACATAG-3′
*Nfe212*-F	5′-TTGTAGATGACCATGAGTCGC-3′
*Nfe212*-R	5′-ACTTCCAGGGGCACTGTCTA-3′

**Table 2:** Phytochemical content and antioxidant activity of *Stahlianthus involucratus.*

Methods	ASI	MSI
TPC (mg GAE/g dried extraction)	9.25 ± 0.25^b^	110.24 ± 1.01^a^
TFC (mg QE/g dried extraction)	0.44 ± 0.03^b^	12.30 ± 0.10^a^
DPPH (mg TE/g dried extraction)	6.86 ± 0.13^b^	21.09 ± 0.34^a^
ABTS (mg TE/g dried extraction)	67.58 ± 1.16^b^	89.62 ± 1.65^a^
FRAP (mg TE/g dried extraction)	7.35 ± 0.24^b^	30.60 ± 0.27^a^

**Table 3:** Characterization of the metabolites of *S. involucratus* by UHPLC-Q-Orbitrap-MS.

	Compounds name	Molecular formula	Observed (*m*/*z*)	RT (min)	Ionization mode
1	4-Pyridoxic acid	C_8_H_9_NO_4_	182.0450	5.064	[M-H]^−^
2	O-Desmethyl-cis-tramadol	C_15_H_23_NO_2_	250.1801	6.352	[M+H]^−^
3	2-(Hydroxymethyl)-6-[(E)-4-(1,2,4-trihydroxy-2,6,6-trimethylcyclohexyl)but-3-en-2-yl]oxyoxane-3,4,5-triol	C_19_H_34_O_9_	451.2184	8.245	[M-H]^−^
4	3-[2-(*β*-D-Glucopyranosyloxy)-4-methoxyphenyl]propanoic acid	C_16_H_22_O_9_	357.1188	9.351	[M-H]^−^
5	Vanillic acid	C_8_H_8_O_4_	167.0839	9.658	[M-H]^−^
6	1,2,3,6-Tetra-O-galloyl-*β*-D-glucose	C_34_H_28_O_22_	787.0996	10.789	[M-H]^−^
7	4-Coumaric acid	C_9_H_8_O_3_	165.0547	11.154	[M+H]^−^
8	Ferulic acid	C_10_H_10_O_4_	193.0496	11.539	[M-H]^−^
9	4-[4-(4-Hydroxy-3-methoxyphenyl)tetrahydro-1H,3H-furo[3,4-c]furan-1-yl]-2-methoxyphenyl hexopyranoside	C_26_H_32_O_11_	565.1692	11.823	[M-H]^−^
10	Astragalin	C_21_H_20_O_11_	465.1044	13.368	[M-H]^−^
11	1,7-Bis(4-hydroxyphenyl)-3,5-heptanediol	C_19_H_24_O_4_	315.1599	13.570	[M-H]^−^
12	Afzelin	C_21_H_20_O_10_	431.0919	14.101	[M-H]^−^
13	2-(8-Hydroxy-4a,8-dimethyldecahydro-2-naphthalenyl)acrylic acid	C_15_H_24_O_3_	251.1648	16.519	[M-H]^−^
14	1-Naphthol	C_10_H_8_O	143.0489	16.863	[M-H]^−^
15	Curcumin	C_21_H_20_O_6_	385.1292	16.963	[M-H]^−^

## Data Availability

The data used to support the findings of this study are included within the article.
